# Anomalous Origin of the Anterior Choroidal Artery Proximal to an Ipsilateral Fetal Posterior Cerebral Artery: Case Report of an Extremely Rare Anatomic Variant and Discussion of its Clinical Implications

**DOI:** 10.7759/cureus.6442

**Published:** 2019-12-22

**Authors:** Tatiana Mamaliga, Andrew C White, Nicolas K Khattar, Mohiuddin Hadi

**Affiliations:** 1 Radiology, University of Louisville, Louisville, USA; 2 Neurosurgery, University of Louisville, Louisville, USA; 3 Radiology/Neuroradiology, University of Louisville, Louisville, USA

**Keywords:** anterior choroidal artery, anatomic variant, cerebral vascular anatomy, neuroendovascular, cta, incidental

## Abstract

The anterior choroidal artery (AChA) supplies important cerebral structures such as the internal capsule, optic tract, and lateral geniculate body. It is normally a branch of the communicating segment of the internal carotid artery (ICA) with significant neurological symptomatology if injured. Typically, the AChA originates distal to the posterior communicating artery (PCoA), but rare variants have previously been described. Such anatomical differences are particularly important entities, especially in the context of neuroendovascular procedures, as inadvertent damage to these arteries can have devastating consequences. In this case report, we present an unusual anatomical pattern of the right AChA arising proximal to an ipsilateral fetal PCA.

## Introduction

The anterior choroidal artery (AChA) serves a critical role during brain development and continues to supply a number of eloquent structures in the adult brain, including the globus pallidus interna, posterior limb of internal capsule, optic tract, parts of the thalamus, tail of the caudate nucleus, cerebral peduncle, substantia nigra, red nucleus, and choroid plexus [1-3]. It is classically described as the terminal branch of the communicating segment of the internal carotid artery (ICA), arising laterally and distally to the posterior communicating artery (PCoA) [2,4-6]. Numerous reports have described variations to the branching pattern, course, size and ultimate vascular territory of the AChA [6-8], but variation in the origin of the vessel distal to the posterior communicating artery is rarely observed. Few reports describe a common origin for the AChA and the PCoA [9], and others report the AChA arising from the middle cerebral artery (MCA) or PCoA. Identifying such variance in the vascular anatomy is especially important during pre-operative to mitigate risks of potentially devastating neurological consequences in the event of an injury. This is the sixth reported case of an AChA originating proximal to the PCoA [4,8,10-12]. All but one of the previous reports of a proximal origin had associated aneurysms [4,8,10-12]. We present a complex vascular pattern with an anomalous origin of the AChA proximal to the PCoA associated with a persistent fetal posterior cerebral artery (PCA).

## Case presentation

**Figure 1:**
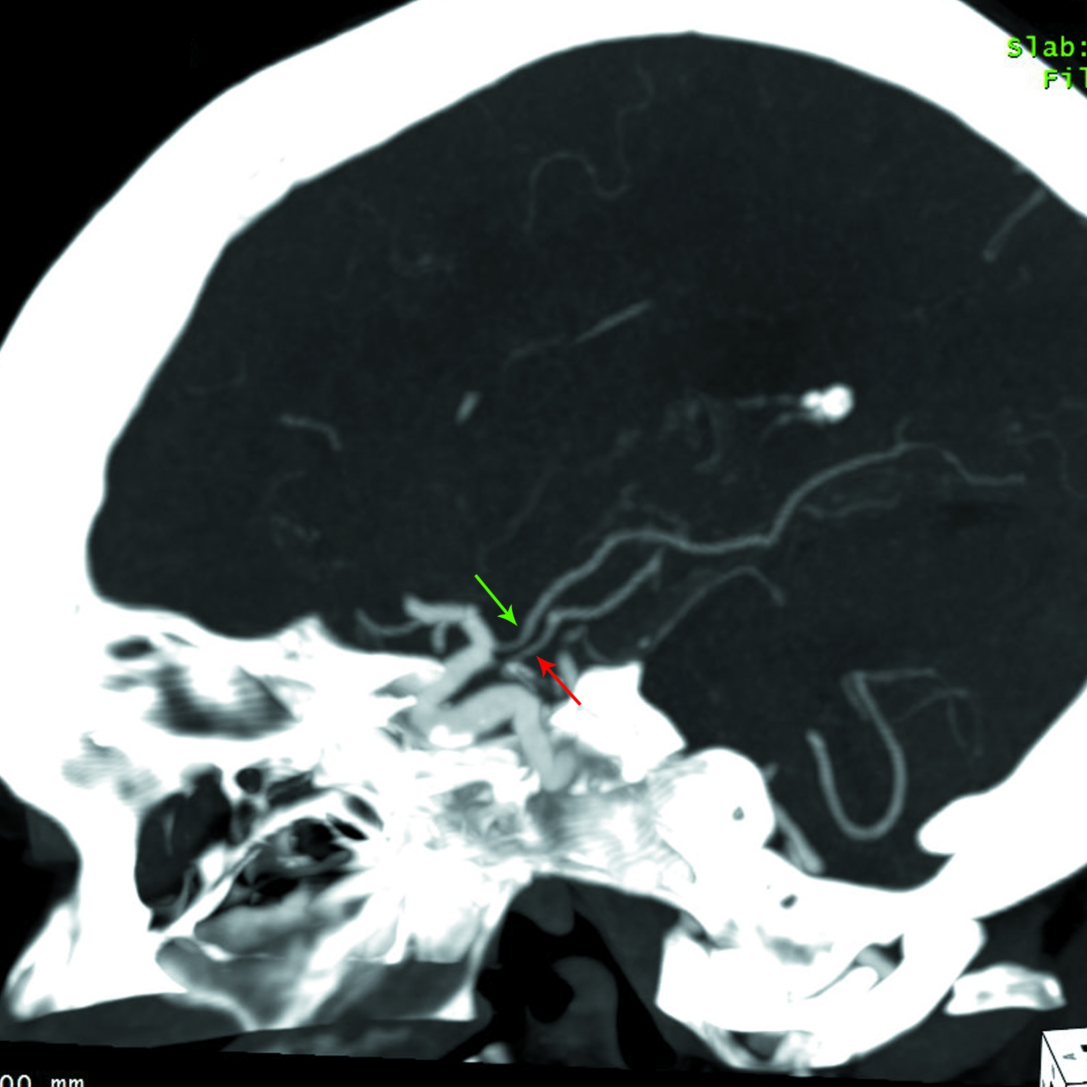
Sagittal MIP reformatted images from CT angiogram of the head showing the course of the right ICA A fetal PCA arises from the posterior aspect of the ICA and has a normal posterior course (green arrow). An additional vessel arising in close proximity but proximal to the fetal right PCA parallels its course until the P2/3 segment, where it ascends, crossing the PCA, and continues to terminate within the choroid plexus in the right lateral ventricle (red arrow). MIP = maximal intensity projection; CT = computed tomography; ICA = internal carotid artery; PCA = posterior cerebral artery

**Figure 2:**
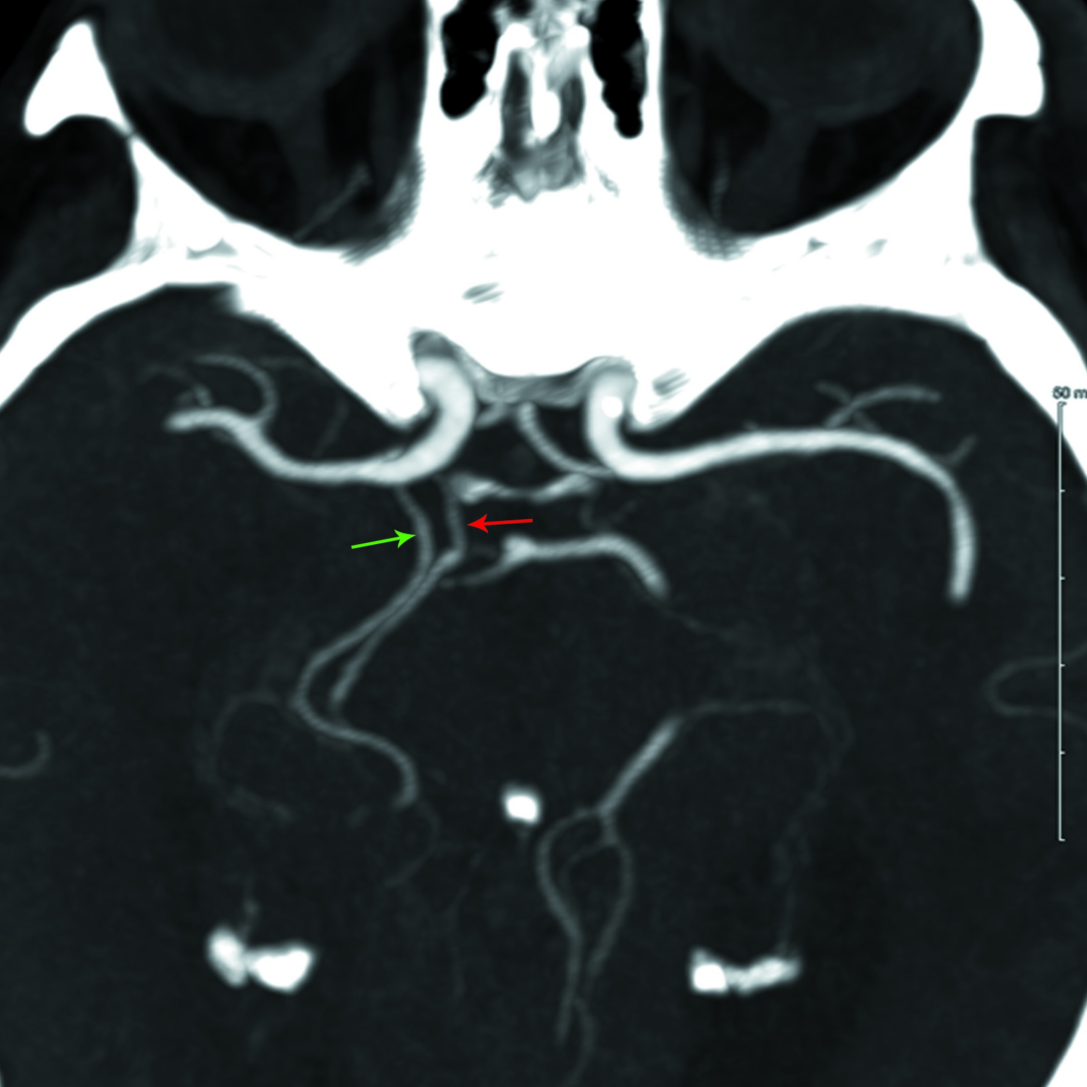
Axial MIP reformatted images from CT angiogram of the head showing two vessels arising from the posterior aspect of the distal ICA The proximal (more medially-oriented) vessel (red arrow) courses posterior, paralleling the fetal right PCA (green arrow) until the P2/3 segment. MIP = maximal intensity projection; CT = computed tomography; ICA = internal carotid artery; PCA = posterior cerebral artery

**Figure 3:**
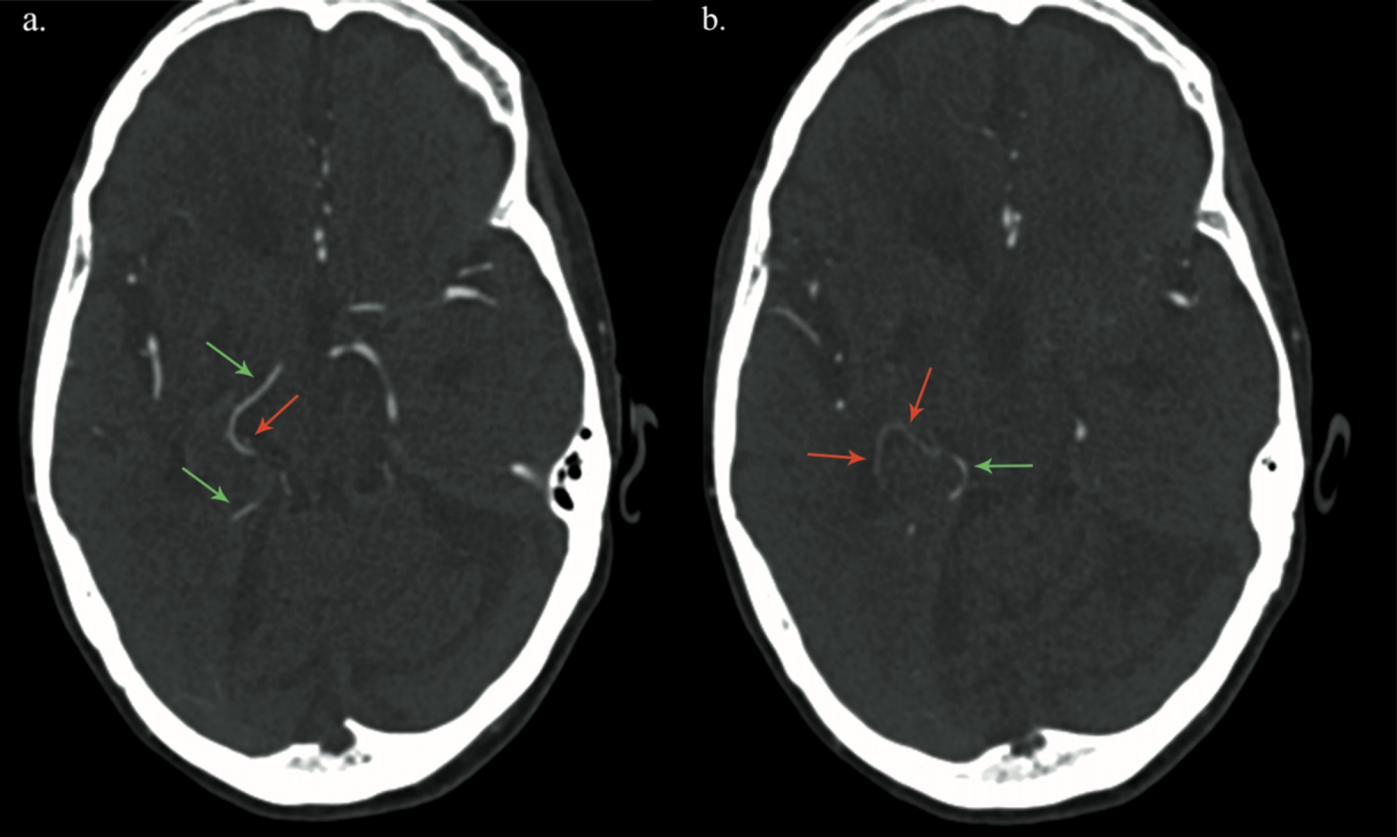
Axial images from CT angiogram of the head through the level of the Foramen of Monroe (a.) and third ventricle (b.) demonstrating the relationship between the fetal right PCA (green arrows) and anomalous AChoA (red arrows) Near the medial aspect of the right side tentorium cerebelli, the anomalous AChoA dives under the P2/3 segment of the fetal right PCA, courses anterolaterally for a short distance, and then ascends to terminate at the choroid plexus in the right lateral ventricle. CT = computed tomography; PCA = posterior cerebral artery; AChoA = anterior choroidal artery

**Figure 4:**
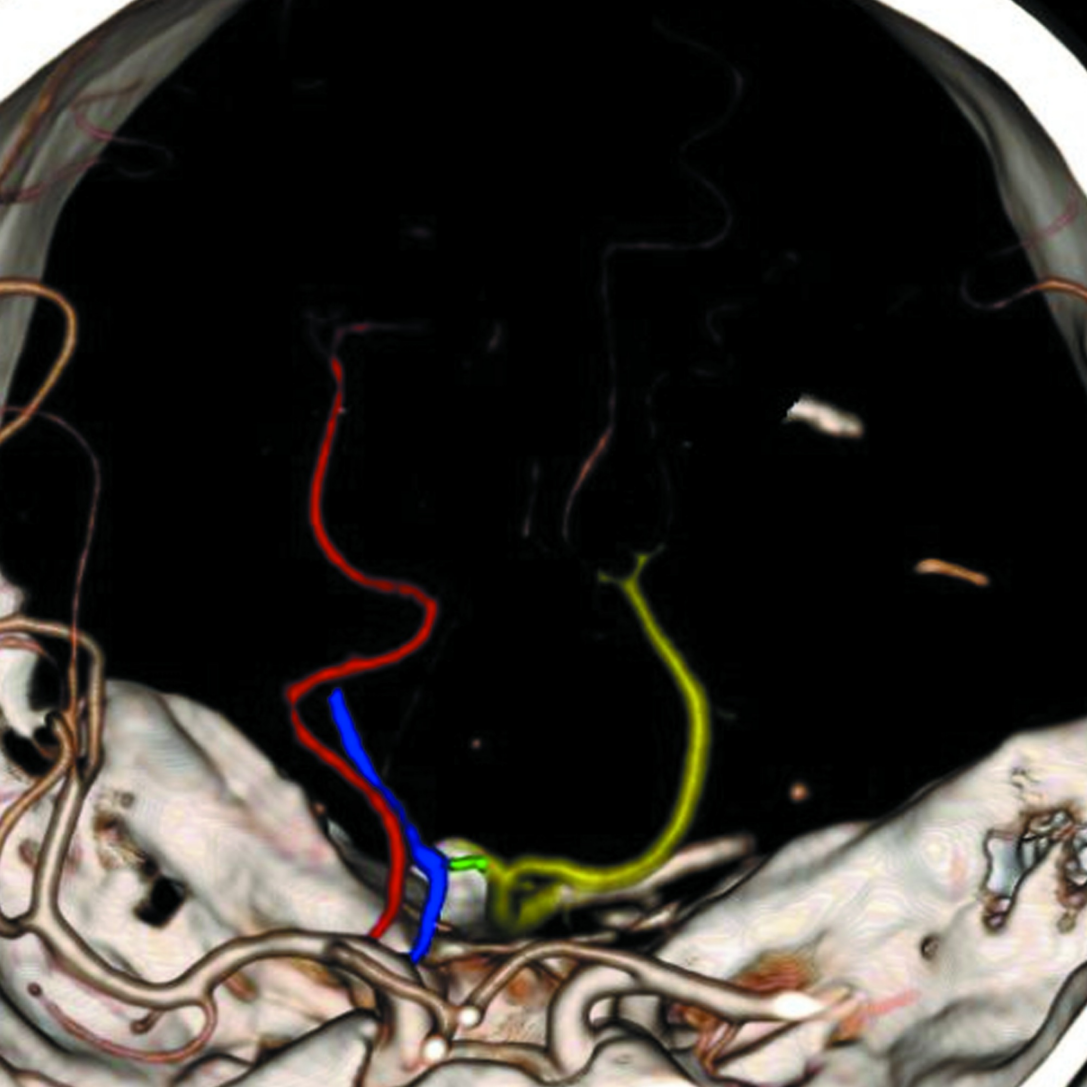
3D volume rendered image with colored vessel overlay showing the fetal right PCA (red), lateral (and distal) to which the anomalous AChoA (blue) arises, forms an anastomosis (green) with a diminutive P1 segment coming from the basilar artery (yellow), and then continues its course to the choroid plexus in the right lateral ventricle PCA = posterior cerebral artery; AChoA = anterior choroidal artery

Our patient is a 62-year-old woman who presented to the emergency department from a skilled nursing facility for evaluation after sustaining a fall from her wheelchair. A noncontrast-enhanced CT of the brain did not show any acute intracranial findings but revealed a chronic infarct in the right MCA territory. As part of the standard emergency department work-up, a CT angiogram (CTA) of the brain and neck was performed which incidentally revealed the unusual variant anatomy of the communicating segment of the right ICA. There was an anomalous origin of the AChA proximal to an ipsilateral fetal PCA, in addition to an anastomosis between the anomalous AChA and the basilar artery at the expected location of a typical P1 PCA segment (Figures 1-4). All the other major arteries of the Circle of Willis were present and demonstrated classic anatomy. The patient was evaluated by the Stroke team and deemed her safe to return to her nursing care facility within the same day after they ruled out a new ischemic stroke.

## Discussion

We present a case of an anomalous AChA originating proximal to the posterior communicating artery, which has previously been reported in a few individual case reports (Table 1), and collectively described as a very rare occurrence in large anatomic series [4,8-11]. The same anatomic series also described a handful of MCA and PCoA origins of the AChA and in one case a common origin of the PCoA and AChA [3,9].

**Table 1:** Case reports describing an anomalous origin of the anterior choroidal artery PCoA = posterior communicating artery; SAH = subarachnoid hemorrhage; AChA = anterior choroidal artery [4,8,10-12]

Case #	Authors, Year	Laterality	Associated Findings
1	Hara et al., 1989	Left	None.
2	Moyer et al., 1992	Left	Multiple scattered aneurysms, including one near the origin of the left PCoA, and SAH from rupture of an aneurysm.
3	Nomura et al., 2000	Left	Unruptured aneurysm at the origin of the left AChA.
4	Nishio et al., 2009	Left	Unruptured aneurysm at the origin of the left AChA.
5	Choi et al., 2012	Left	Multiple scattered aneurysms and SAH due to ruptured aneurysm arising from an ipsilateral fetal type PCoA.

Knowledge regarding anomalous manifestations of the AChA is extremely important in open surgical and endovascular procedures to prevent devastating neurological consequences [2,13]. The AChA has an extensive branching pattern that supplies a number of critical structures, including the globus pallidus interna, posterior limb of the internal capsule, optic tract, parts of the thalamus, tail of the caudate nucleus, cerebral peduncle, substantia nigra, red nucleus, and choroid plexus [1,2]. Hyun et al. described a case of coil embolization of an aneurysm associated with an anomalous AChA that led to acute infarction of the internal capsule despite continued patency of the AChA post-surgically [2]. Various studies described the most common neurologic deficits associated with AChA infarction. Derflinger et al. reported the symptoms of 30 patients, which included: hemiparesis (100%), dysarthria (70%), dysphagia (40%), hemisensory abnormalities (37%), dysphasic symptoms (20%), and gaze-induced nystagmus (7%) [14]. In a series of 112 patients, Ois et al. also describes symptomatology associated with AChA infarcts and showed contralateral motor weakness (77.6%), sensory dysfunction (75.8%), aphasia (16.9%), contralateral hemianopia (14.2%), hemiparesis (12.5%) and confusion (2.67%) [15]. Furthermore, patients with AChA strokes with perfusion deficits typically showed the progression of the disease and worse neurological signs due to ischemic zone expansion and worsening of the initial perfusion deficit [16]. Therefore, unfamiliarity with a variable anatomy of the AChA before performing a surgical or embolic procedure such as for the treatment of a stroke/cerebrovascular disease/aneurysm could potentially have devastating neurological consequences.

Currently, there does not seem to be an explanation from the developmental point of view for the anomalous origin of the AChA [8,10]. The embryological origin of the AChA is well described from the rostral division of the ICA, which also gives rise to the anterior cerebral and middle cerebral arteries. The caudal division of the ICA gives rise to the PCoA and anastomoses with the PCA [17]. Nishio et al. have proposed the idea of a mutation causing the anomalous origin of the AChA [11].

Fetal PCA is a common variant with significant implications on the vascular anatomy and brain perfusion and should be carefully evaluated while planning surgical and endovascular procedures. A fetal PCA occurs in 20% to 30% of the population on CTA [18]. In the presence of a fetal PCA, the basilar artery is no longer the main supplying vessel to the ipsilateral PCA. Instead, the ICA continues to assume that function and gives rise to the PCA, resulting in either no or a small connection between the PCA and the basilar artery, also termed a hypoplastic P1 segment [19]. Moreover, since the connection between the PCA and the basilar artery is either lost or becomes negligible through a diminutive P1 segment, the ipsilateral ICA-PCoA segment receives higher blood flow and therefore has increased pressure within it [20]. These changes have important implications when treating stroke patients.

## Conclusions

An anomalous origin of the AChA is an important anatomical variant that radiologists, neuro-interventionalists and neurosurgeons alike should be cognizant of while planning interventions to prevent devastating neurological consequences. Further evaluation with digital subtraction angiography should be undertaken if such anomalous vasculature is detected on CT angiography. Such additional interventions would also provide a better understanding of the brain territory that the anomalous variant supplies.
